# ICT Adoption and Booming E-Commerce Usage in the COVID-19 Era

**DOI:** 10.3389/fpsyg.2022.916843

**Published:** 2022-06-10

**Authors:** Tong Zou, Ali Cheshmehzangi

**Affiliations:** ^1^Department of Architecture and Built Environment, University of Nottingham Ningbo China, Ningbo, China; ^2^Network for Education and Research on Peace and Sustainability (NERPS), Hiroshima University, Hiroshima, Japan

**Keywords:** COVID-19 pandemic, e-commerce, ICT adoption, digital technologies, online, COVID-19 era

## A Brief Introduction to E-Commerce Usage in the COVID-19 Era

With the rise of the digital economy, digitalization has fundamentally transformed the way we live and work [Organization for Economic Co-operation Development (OCED), 2019]. Since the outbreak of the COVID-19 pandemic in late 2019, people have been increasingly relying more on digital technologies due to pandemic prevention and control strategies such as lockdown, social distancing, enclosure of restaurants and malls, etc. COVID-19 has been intensively transforming the world's trends, people's behavior, nature of business, and daily life (Bhatti et al., 2020). Meanwhile, it has been acting as a catalyst or accelerator of this new digital revolution in the COVID-19 Era, especially for the rapid adoption of e-learning and e-commerce globally.

Both direct and indirect impacts of COVID-19 have forced people to purchase goods online. Meanwhile, traditional producers and traders have also moved their business online regarding the declining shares of conventional trade and the unstable markets due to the COVID-induced supply chain disruptions, resource shortage, lack of human capital, declined customer demand, etc. The World Trade Organization (WTO; Abdelrhim and Abdullah, 2020) states that it is the exact time for e-commerce to rescue the global economy from the COVID-19 and that it is to interfere with vigor and energy and demonstrate e-commerce of its prominence and effectiveness in the field of trade and online shopping.

## Methodology

**Figure 1:**
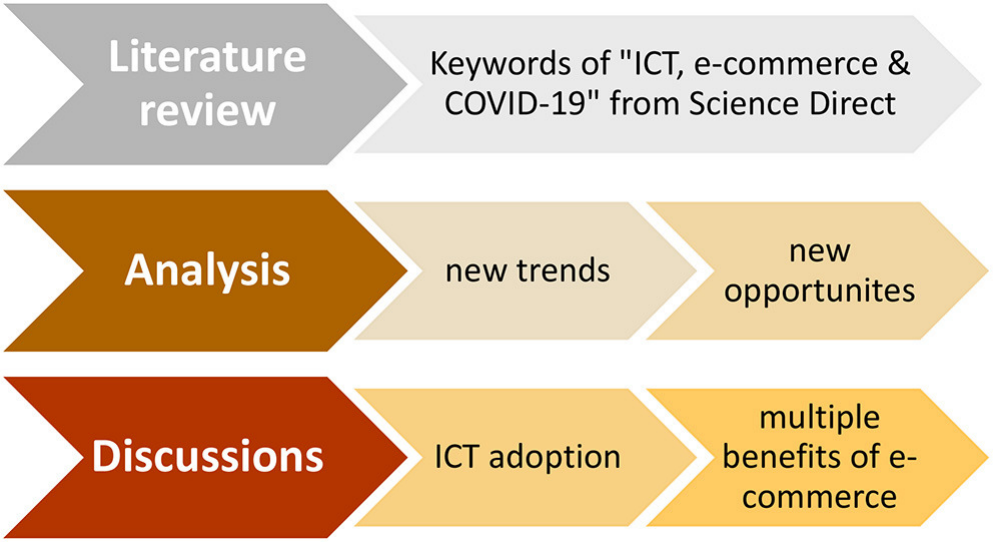
Methodology flow.

This study is conducted in three steps. First, the literature review is conducted systematically to review existing recent studies related to the nexus between ICT, e-commerce, and COVID-19, evaluating recent research trends/focus in the area. Second, we follow up the literature review by analyzing the new trends and new opportunities. Here, relevant studies on this topic are selected to support the background basis and inform the content for further discussions. Third, discussions on ICT adoption and multiple benefits of e-commerce are provided based on the results of the first two steps. Figure 1 summarizes the methodology flow in three steps.

## Literature Review

The impacts of the COVID-19 pandemic have been growing well recognized globally across all industries among all groups of people, and disruptions of the global supply chain have created an excellent chance to explore the promising potential of e-commerce as a mode of future trade. This results in booming e-commerce and leads to accelerated ICT adoption driven by digital innovation that continues to rise sharply in the pandemic era. A study using panel data in Japan shows that e-commerce purchases of grocery goods witnessed a dramatic increase during the pandemic, followed by machinery/PC, books, and software, while young females are found to be the most frequent user of e-commerce (Kawasaki and Wakashima, 2022). Moreover, no significant correlations were identified between e-commerce usage and regional characteristics like population density, city size, and transportation means for shopping. At the same time, convenience and utility are the main reasons people choose e-commerce (Kawasaki and Wakashima, 2022). However, unlike telecommuting during the COVID-19 pandemic, e-commerce does not always provide convenience to people regarding social wellbeing and social sustainability. Research reveals that the booming food e-commerce elevated by the pandemic in Canada barely offers extra convenience to people who already have sufficient food access while keeping the status quo or even declining food access to vulnerable and socially disadvantaged groups (Music et al., 2022). This reflects that COVID-19 is not only a powerful catalyst for e-commerce (Beckers et al., 2021) but also for the growing digital divide as a multiplier of deepening digital barriers as never before.

Another study shows that technical-related enablers (e.g., ease of operation of e-commerce platform, awareness of e-commerce benefits, sufficient security, and availability of internet facilities) are the most significant factors of e-commerce adoption in the pandemic. They have the highest influence on the delivery time (Cheba et al., 2021). This finding implies the importance of reducing the digital divide and supporting digital infrastructures in enhancing firm performance in terms of time efficiency. Furthermore, it is also suggested that pandemic-related enablers (e.g., provision of protective health equipment, development of economic recovery programs, adherence to policy change, the introduction of new service projects) can partially mediate the relationship between firm performance and other e-commerce adoption enablers. This correlation involves institution-related enablers (e.g., competitive business environment, sufficient trust among stakeholders, etc.), firm-level enablers (e.g., highly skilled employees, effective communication flow, sufficient budgetary allocations, etc.), and technical-related enablers as well (Cheba et al., 2021). Similarly, several types of factors in the urban e-commerce market are identified. These include internet and mobile access (e.g., individuals use smartphones, internet bank account penetration), macroeconomic conditions (e.g., domestic market size, purchasing power), social dimensions (e.g., trustworthiness and confidence, legal frameworks' adaptability to digital business models), urban e-commerce activity (e.g., internet use for selling goods or services), and environmental conditions (e.g., carbon dioxide from transportation and storage; Juliet Orji and Ojadi, 2022).

On the other hand, it is also argued that the pandemic offers excellent potential for improving the three components of e-retail accessibility (i.e., individual, web infrastructure, and temporal components). At the same time, it may also create difficulties and burdens for the traditional retailers if they feel difficult and/or fail to transform and digitalize their business from offline to online due to t lack of digital literacy, skilled professionals (Beckers et al., 2021), high economic cost and possible long payback period, especially during this special time of the pandemic.

At the same time, the reoccurrence of pandemic outbreaks with continuous pandemic control and prevention policies like lockdowns and social distancing rules might speed up the evolution progress of multi-channel retail and the channel integration role of offline shops, resulting in urban-retail landscape transformation (Nanda and Xu, 2021). This calls for relocations and distributions of traditional offline shops' business and functions through multi-channels (Nanda and Xu, 2021). Moreover, evidence illustrates that the connections between the perceived effectiveness of e-commerce platforms, economic profits, and sustainable consumption can be moderated by pandemic fear (Tran, 2021). It is also found that the impacts of the COVID-19 pandemic on people's sense of utilizing e-commerce are much more complicated. The situation is due to diverse alterations triggered by the pandemic in the need mechanism of consumers of e-commerce platforms (Inoue, 2022), suggesting further investigations on examining the mechanisms and other factors such as user innovations, consumer behaviors, and types of e-commerce platforms. For instance, even though there have been growing studies investigating the changes in the e-commerce market since the start of the pandemic pop-up in the literature (Juliet Orji and Ojadi, 2022), factors and determinants of consumers' behaviors regarding online buying and e-commerce are still uncertain (Guthrie and Fosso-Wamba, 2021).

In short, the current literature on ICT, e-commerce, and COVID-19 is continuously growing with the pandemic-accelerated booming e-commerce and digital innovations. However, there are still several gaps regarding this topic. First, the uncertainties and mechanisms associated with both internal and external factors affecting ICT adoption and e-commerce usage during and after the pandemic are underexplored. Second, investigations and analyses on the barriers, burdens, and indirect and negative impacts of ICT adoption and e-commerce are limited. Third, studies and innovations for possible strategies and sol to offset negative impacts, maximize benefits, mitigate the externalities, and reduce the growing digital divides empowered by the nexus between e-commerce, ICT adoption, and the pandemic of COVID are needed.

## New Trends and Opportunities: Exploring the New Norms

It is argued that “E-Commerce” has become the driver of digital innovation over the last 10 years (Statista, 2022a). In 2020, global e-commerce sales increased by 19% due to the COVID-19 pandemic (Statista, 2022a). According to the e-commerce report 2021 (Statista, 2022a), the United States (US$537.7 billion), China (US$1,343.5 billion), and Europe (US$460.5 billion) are the three major e-commerce markets. China was the largest market in 2020 and will be staying the first with the greatest predicted growth rate of the three major regions (i.e., Compound Average Growth Rate of 8.2%). The report identifies a growing trend in purchasing power moving from the US and Europe to China and Southeast Asia (Statista, 2022a). Besides the impacts of COVID, the primary underlying reason might be that some digital divide barriers among people living in the global south have been gradually reduced. It has enabled Asian consumers to access e-commerce due to rising purchasing power and internet penetration, particularly on mobile devices. Moreover, the benefits and convenience brought by e-commence have fostered people's preferences and behaviors toward its usage. One study reveals that only 48% of consumers are now refusing to go brick-and-mortar shopping in crowded places, and this number only increased by 16% (i.e., 64%) after receiving the COVID vaccine (Bhatti et al., 2020). This implies the trend of growing e-commerce consumers is unlikely to be changed even in the post-COVID-19 era.

On the other hand, e-commerce in the COVID-19 era also brings SMEs (small and medium enterprises) more opportunities, especially in the food sector. Food is the basic need for sustaining people's lives, while individuals' food security level faces multiple challenges in the COVID-19 era. Among the five segments of e-commence, the global e-commerce sales in “Food and Personal Care” increased by 29% in 2020, followed by toys, hobby and DIY (21%), electronics and media (19%), furniture and appliances (17%), and fashion (14%; Statista, 2022a). It is commonly recognized that e-commerce can bring SMEs and larger companies alike equal access to global markets. It can also support vertical coordination processes in the food supply chain (Zeng et al., 2017). ICT-mediated third-party commercial and retail platforms are the vehicles bridging SMEs and the benefits of e-commerce together. Various advantages of those platforms have been well recognized (Statista, 2022b), including huge network flow, massive views and exposure, low cost of platform expansion, and high speed of platform expansion.

In addition, it is known that besides internet penetration, smartphone penetration plays a significant role in e-commerce. With an increasing trend of mobile devices usage, more social media and news consumption have switched to mobile-only platforms (Statista, 2022a). E-commerce goes beyond selling products and delivering services by using multiple ICTs to promote and popularize their products. It widens marketing channels and attracts targeting costumers through direct communication, easier exchange of information, improved interaction, increased mobile accessibility, etc. In short, it can be argued that there is a rising trend in the adoption of ICT-mediated commercial platforms and e-commerce among SMEs with a more intensive focus on mobile e-commerce in the COVID-19 era.

The dramatic growth of e-commerce in the COVID-19 era also brings elevating negative impacts in multiple areas. The major issue is the waste generated by packaging, followed by rising traffic pressures and associated greenhouse gas emissions (Tokar and Jensen, 2021). The most visible negative effect of e-commerce is packaging and overpackaging waste, directly connected to GHGs emissions from the associated material and energy use. It is claimed that sustainability in e-commerce can be accomplished by promoting cellulose-based materials for packaging. It is one of the most naturally abundant and most vital renewable materials with the minimum environmental impacts (Escursell and Llorach-Massana, 2021). Concerning the implications of e-commerce associated packaging and overpackaging, the current trend is to promote circular packaging, search for zero-waste packaging materials and optimize package distribution through effective policies, regulations, technologies, strategies, and solutions (Escursell and Llorach-Massana, 2021). Researchers and scholars hold different opinions regarding the impacts of e-commerce associated with traffic and emission issues. It is claimed that the adverse impacts of the growth in delivery trucks have been counterbalanced by the benefits of fewer consumers on the road (Peng, 2019; Tokar and Jensen, 2021). While some studies found that the negative influences of delivering e-commerce packages have been continuously increasing (Zhou, 2014; Laghaei and Faghri, 2016).

Lastly, there has been a growing trend of declining conventional commerce with fewer brick-and-mortar places; meanwhile, the share of e-commerce will continue to grow and become the dominant way of business in the COVID-19 era. For instance, over 9,300 retail locations from various categories of products were closed by their parent companies in 2019, while more than half of shopping malls are expected to be closed by 2021 (Tokar and Jensen, 2021). This trend has fueled the unprecedented rising digital innovations in e-commerce such as live streaming (Hu, 2020), cloud computing (Almarabeh, 2019), artificial intelligence and machine learning (Patil, 2019; Zhang and Pee, 2021), drone deliveries (Aurambout and Gkoumas, 2019), etc. Meanwhile, this fast pace of digital innovation and e-commerce expansion also brings multiple issues and challenges like privacy concerns about customers' information (Maseeh et al., 2021), cyber fraud (Hamirani, 2020), and issues related to governance and regulation enforcement [Organization for Economic Co-operation Development (OCED), 2019]. For instance, regulatory approaches and governmental enforcement of government have been challenged by digital technologies [Organization for Economic Co-operation Development (OCED), 2019] due to two primary reasons. First, the digitalization of trading and/or e-commerce has blurred the conventional definition of markets while surpassing administrative boundaries nationally and globally. Secondly, digital technologies develop much quicker than the regulation or social structures governing them. In a word, e-commerce, technological advances, and digital innovations have brought various benefits and opportunities to our life and economy, while the associated negative impacts and/or side effects must be dealt with more carefully to make the best potential of e-commerce.

## Discussions: ICT Adoption and Multiple Profits of E-Commerce Boosted by the COVID-19 Pandemic

E-commerce is considered a specific and in-depth application of current and emerging ICT to conduct business (Li et al., 2021). Its relatively low entry threshold reduces some social inequality barriers and offers opportunities to disadvantaged populations and/or marginalized groups, including SMEs, rural regions, vulnerable people, etc. For example, e-commerce has increased farmers' employment channels and livelihood choices, further benefiting the economy and development of rural and remote areas with poverty alleviation (Zeng et al., 2017; Li et al., 2021). ICT adoption can reduce reasonable costs for SMEs and improve competition, knowledge management, and access to robust business information. It could help efficiently manage materials and resources and broaden market and trade liberty (Al Busaidi and Bhuiyan, 2019). Furthermore, from customers' perspectives, e-commerce for SMEs can enhance service availability, communication with customers, information exchange, sales, and productivity following customers' demands (Alzahrani, 2018).

The growth of food e-commerce in the COVID-19 era is the most significant. As mentioned earlier, the COVID-19 pandemic has become an accelerator of the process for people to adopt ICT and e-commerce and as a catalyst for their associated impacts, benefits, and side effects. It is found that the proportion of confirmed infected COVID-19 cases rises the likelihood of consumers buying their food online, and this link is found to be more significant for young people having a lower perceived risk of online shopping and living in big cities (Gao et al., 2020). Moreover, it is suggested that the marketplace digitalization and people's online shopping habits gained during COVID-19 will remain the same and eventually might trigger structural changes to business operation and consumption patterns (Guthrie and Fosso-Wamba, 2021). This is likely as people keep their adapted purchasing behaviors once the pandemic ends (Kim, 2020; Sheth, 2020), such as those witnessed in China in 2002–2003 (Clark, 2016) during the SARS pandemic.

It is argued that the COVID-19 pandemic holds sufficient opportunities for growth in three components of e-commerce accessibility (Beckers et al., 2021). For individual components, e-commerce in the COVID-19 era attracts local consumers due to travel restrictions, social distancing policies, lockdowns, and lack of long-distance delivery services. Secondly, the digitalization of the marketplace pushes enterprises (especially SMEs) to open online businesses and increases their Web infrastructure component. It is claimed that COVID-19 boosts a growth rate of 30% in the number of local retailers with e-commerce services (Beckers et al., 2021). With the extra aid of social media, retailers gain opportunities to advertise and promote their products online. They can attract, interact, and communicate with more customers directly and improve their management and products based on customers' feedback. The last element of e-commerce is the temporal component. It is linked to delivery distance subject to dominating local e-consumers in the COVID-19 era (Beckers et al., 2021). It is known that “*the last mile*” is the least cost-efficient and most time-consuming part of the whole supply chain (Nanda and Xu, 2021; Tokar and Jensen, 2021). With more local consumers and short distances, 25% of the retailers use a sustainable delivery mode while 23% apply a within-24-h delivery mode (Beckers et al., 2021). This implies that COVID-19 has boosted the adaptation and utilization of ICTs and e-commerce in local retailers/enterprises via the enhancement of e-commerce accessibility and may further support localization and diversification of enterprises. At the same time, it contributes to supply chain resilience toward sustainable e-commerce.

Furthermore, we highlight the increase in the food segment of e-commerce. Food is essential to sustain people's lives while the source/supplier/producer of food covers many individuals/retailers/enterprises. Before the COVID-19, some people could get their food directly from restaurants, while cooking fresh food was not a necessary option. However, the pandemic control and prevention strategies, like restaurants closures and lockdown, have dramatically changed people's accessibility to food, forcing people to cook their own meals at home. The situation has caused a sharp increase in fresh food demand. Yet, most fresh food growers/suppliers/retailers are SMEs or even individual framers. Due to their limited digital literacy and infrastructure, they are more likely to face multiple digital divide barriers to switching to e-commerce by themselves. Hence, ICT-mediated e-Commence platforms connecting SMEs and customers have been springing up, while fresh food e-commerce platforms have experienced growth and unprecedented opportunities brought by COVID-19. In China particularly, the active online customers of fresh food e-commerce have increased by 70.30%, from 48.94 million in 2019 to 69.61 million in 2021, while the gross merchandise value (GMV) has increased by 46.91%, from 163.9 billion RMB in 2019 to 239.6 billion RMB in 2021 (Statista, 2022b). Therefore, the pandemic has speeded up the growth of ICT-mediated third-party e-commerce platforms as well as the volume and population of both e-Retailers and e-Consumers, especially in fresh food e-commerce.

Lastly, we summarize the findings here, including new trends in the COVID-19 era, multiple benefits and profits boosted by the COVID-19 pandemic, and future research directions covering primary aspects and topics. Table 1 below summarizes these findings.

**Table 1:** Summary of findings related to new trends, multiple benefits and profits, and future research directions.

**New Trends**	• A growing trend in online purchasing power moving from the global north to the global south (i.e., from the US and Europe to China and Southeast Asia); • A growing trend in ICT-mediated third-party commercial and retail platforms (i.e., digitalization of marketplace), with a more intensive growth in food e-commerce; • A rising trend in the adoption of ICT-mediated commercial platforms and e-commerce among SMEs (i.e., integration of B2C [Business to Customer] and O2O [Online to Offline]), with a more intensive focus on mobile e-commerce; • A declining trend in conventional brick-and-mortar marketplaces; • A rising trend in recognizing the negative impacts of e-commerce with considerations of fostering sustainable e-commerce and promoting digital innovations; • A trend where people maintain their purchase behavior gained during the pandemic (i.e., online shopping).
**Multiple benefits and profits boosted by the COVID-19 pandemic**	• Acceleration of adopting ICT and e-commerce and business digitalization; • Catalyzation of e-commerce associated impacts, benefits, and side-effects, as well as digital innovation; • Reduces some social inequality barriers and offers opportunities to disadvantageous populations and/or marginalized groups; • Support SMEs, rural economy, local business environment, and diversification of enterprises; • Improve e-commerce accessibility, supply chain resilience, and even distribution of resources; • May eventually contribute to positive peace and sustainability if related trade-offs, specific digital divide barriers, and associated negative impacts can be fully considered and adequately addressed.
**Future research directions**	• E-commerce governance and regulation enforcement (e.g., customer privacy, cyber fraud) • Reduce digital divide barriers for socially disadvantaged/marginalized groups [e.g., inclusive technology designs for the elderly and children, ageism-free EICT (Everyday Information and Communication and Technology) settings Köttl et al., 2021] • E-commerce associated negative impacts such as improving logistic systems, smart warehouses, AI-optimized distribution systems, reducing packaging/sustainable packaging, resolving the last mile issue [e.g., implementation of mobile warehouses for last-mile delivery Srivatsa Srinivas, 2021], etc. • Education of rural e-commerce leaders Li et al., 2021. • Application of blockchain technology in e-commerce to improve the four TRs (i.e., TRaceability, TRacking, TRansparency, TRrust) for decentralization Centobelli et al., 2021; blockchain-based sustainable supply chains and expand its implementation to SMEs Lim et al., 2021; blockchain-based systems of cross-border supply chain management Liu, 2020

## Author Contributions

AC has structured and developed the paper. TZ has completed the literature review and worked on discussions under AC's supervision. All authors contributed to the article and approved the submitted version.

## Funding

AC acknowledges the National Natural Science Foundation of China (NSFC) for the provision of funding for project number 71950410760. He also acknowledges the Ministry of Education, Culture, Sports, Science and Technology (MEXT), Japan Government, and the Network for Education and Research on Peace and Sustainability (NERPS), Hiroshima, Japan.

## Conflict of Interest

The authors declare that the research was conducted in the absence of any commercial or financial relationships that could be construed as a potential conflict of interest.

## Publisher's Note

All claims expressed in this article are solely those of the authors and do not necessarily represent those of their affiliated organizations, or those of the publisher, the editors and the reviewers. Any product that may be evaluated in this article, or claim that may be made by its manufacturer, is not guaranteed or endorsed by the publisher.
